# Design of a corporate financial crisis prediction model based on improved ABC-RNN+Bi-LSTM algorithm in the context of sustainable development

**DOI:** 10.7717/peerj-cs.1287

**Published:** 2023-04-26

**Authors:** Yi Zhao

**Affiliations:** School of Management, Wuhan University of Bioengineering, Wuhan, Hubei, China

**Keywords:** Bi-LSTM, Development, RNN, Sustainable financial crisis prediction

## Abstract

In the context of sustainable economic development, while economic globalization brings new vitality to the company, it also makes the company face an increasingly severe external environment. The managers have to shift their focus to capital market investment. The excessive pursuit of investment benefits can easily lead to decision-making errors, resulting in a financial crisis for the company, and even may be forced to delist in severe cases. This article proposes a financial crisis prediction model based on Artificial Bee Colony—recurrent neural network (ABC-RNN) and bidirectional long short-term memory (Bi-LSTM) company with a characteristic attention mechanism. We combined ABC-RNN with Bi-LSTM to extract more temporal feature vectors from financial data. Then we introduced a feature attention mechanism to extract better depth features from financial data; the ABC algorithm is introduced to optimize the weight and bias of RNN to improve the reasoning speed and accuracy. The experiment shows that the prediction accuracy and recall of the model on the test set have reached 88.94% and 88.23%, respectively, which has good prediction ability. The outcome of this research helps the company to prevent and deal with the financial crisis in time and promote the sustainable development of the market economy.

## Introduction

Implementing the sustainable development strategy is conducive to promoting the unity of economic and social benefits, transforming economic growth from extensive to intensive, and coordinating economic development with population, resources and environment. At the same time, implementing this strategy is also conducive to the economy’s sustainable, stable and healthy development, thereby improving people’s living standards and quality. In recent years, with the steady progress of the sustainable development strategy, major companies have risen rapidly, bringing full economic vitality to national development. However, with the vigorous development of major companies, their high growth and high-risk characteristics are becoming increasingly prominent. Due to problems such as increased requirements for technical iteration, weak solvency, and unstable cash flow, the company is more likely to have potential financial risks or even financial crises. Therefore, relevant companies are in urgent need of targeted financial crisis prediction models to help companies predict whether there are serious financial risks in advance (Brown, Pelosi & Dirska, 2013), thus stabilizing market expectations and supporting the sustainable development of the economy.

With the rapid development of artificial intelligence and deep learning technology, the artificial neural network has demonstrated superior performance in financial crisis prediction (Caro et al., 2020). Recent research shows that recurrent neural network (RNN) has a more vital ability to extract text feature vectors (Tan et al., 2015). Xu, Michael & Stephen (2015) achieved good experimental results using RNN, but their use of traditional RNN structures did not limit vector features, resulting in the inability to transmit deep features continuously. To alleviate this problem, researchers have proposed an LSTM model with long and short-term memory and an improved Bi-LSTM model (Monti et al., 2022). However, using manually extracted features as input in LSTM and Bi-LSTM still fails to achieve good results. Compared with manually extracted feature vectors, word embedding word vectors can better perform feature representation (Sugawara et al., 2016) and can often achieve better financial crisis prediction results when combined with RNN. Yao & Zheng (2016) combined Bi-LSTM and word embedding for training, which can better segment text, but the feature representation ability of word embedding still has some limitations on the classification effect of the model.

To solve the problem of poor prediction effect when the amount of data is large, some researchers proposed combining MLP and CNN to achieve good results. However, in MLP, the order of word vectors is ignored by the network, which affects the accuracy of prediction due to the lack of location information. Although CNN can obtain the order within the range of central features through convolution kernel when extracting features, due to the lack of global information, it is still unable to improve the accuracy of financial crisis prediction significantly.

To further improve the accuracy of the company’s financial crisis prediction, this article proposes an ABC-RNN+Bi-LSTM company’s financial crisis prediction model with a characteristic attention mechanism. The experiment shows that the model presented in this article can accurately predict the company’s financial situation. The work of this article is as follows:

(1) Combine RNN with Bi-LSTM to extract more temporal feature vectors from financial data.

(2) Introduce a feature attention mechanism to extract more high-quality features from financial data.

(3) The ABC algorithm is introduced to optimize the recurrent neural network’s initial weight and bias to improve the model’s reasoning speed and accuracy.

The rest of the article is organized as ‘Related Work’ discusses the related works, followed by financial crisis prediction in the ‘Corporate Financial Crisis Prediction Model’. Experimental design is discussed in ‘Experiment’ and finally, ‘Conclusion’ concludes the work.

## Related Work

In the early research work from the 1960s to the 1980s, the solution of text classification mainly relies on statistics and calculating the similarity between words appearing in the text. The TF-IDF (Luhn, 1958) algorithm measures the degree of correlation between a word in the text and the overall text. It determines the degree of correlation between the word and the topic by comparing the number of occurrences of words in the entire text. The smaller of difference, the more relevant this word is to the theme. The vector space model (Salton, 1975) maps the frequency of occurrence of the same words in different texts into a vector space and uses this vector as a basis for comparing text similarity. However, most of these early methods require tedious feature annotation, which consumes time and energy. Moreover, these methods do not account for the linguistic features of text data and are difficult to learn the deep semantic features of text information. Therefore, the effect of classification presents a bottleneck that is difficult to cross.

With the introduction of Word2vec (Jang, Kim & Kim, 2019), textual information, which is difficult for computers to understand, was converted into numerical vector information. After deep neural networks were proposed, extracting deep semantic features from text data became possible. The bottleneck of text classification was broken by combining Word2vec and deep neural networks. Currently, the neural network approaches are the main methods for processing text data using deep learning. Dynamic CNN (Kalchbrenner, Edward & Phil, 2014) is a convolutional neural network first applied to text classification, which captures textual information hidden in the semantic feature graph by maximum dynamic pooling. Conneau et al. (2016) applied the idea of residual networks to a deep neural network model for processing textual information to obtain text features hidden in deep layers. This type of convolutional neural network ignores temporal information due to the characteristics of the network, although it can extract spatial information very well. LSTM mimics the human state during memory and proposes three gates with unique structures that allow the network to know which information flowing through should be remembered and which should be forgotten. The gated cyclic cell network (Chen & Zhanzhi, 2021) reduces some non-essential operations in the three special gates and improves the accuracy and computational speed.

In contrast, the gated broad learning systems (G-BLS) proposed by Du, Chi-Man & Philip (2020) yielded better results with less arithmetic power. The above LSTM and variants of the LSTM only consider the flow of information from front to back but without assuming that subsequent words are needed in textual information to predict previous words. Sulthana, Jovith & Jaithunbi (2021) addressed this problem by combining a front-to-back LSTM and a back-to-front LSTM to achieve better text classification results.

The Artificial Bee Colony (ABC) algorithm is one of the swarm intelligence optimization algorithms, which mainly simulates the honey collection behavior of bees. The algorithm is simple in structure, easy to implement, and has few parameters, so it has attracted many scholars’ attention and research. Its proposal has solved the problem that the model needs to set parameters repeatedly during training and the gradient disappears, further improving the reasoning speed and generalization ability of RNN and LSTM models (Sreenu & Naidu, 2022). However, the ABC algorithm also has some common problems with evolutionary algorithms. For example, the standard ABC algorithm has some issues, such as slow convergence speed and low accuracy (Yao & Zheng, 2016).

## Corporate Financial Crisis Prediction Model

**Figure 1:**
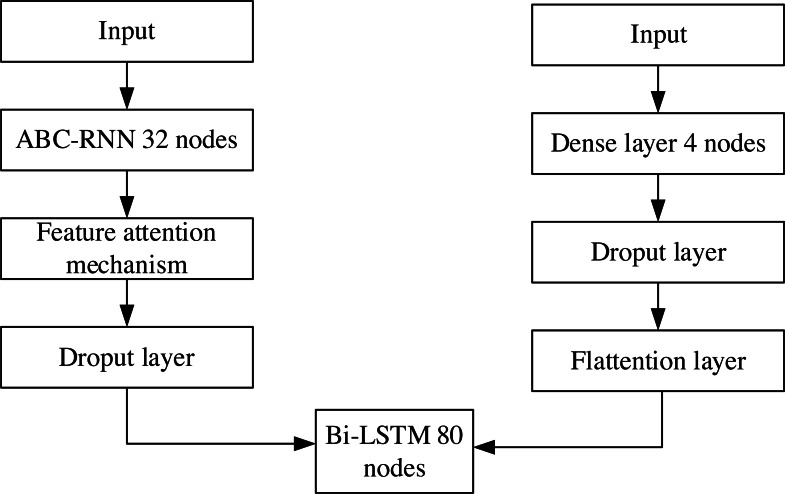
Corporate financial crisis forecasting model framework.

The proposed framework of ABC-RNN+Bi-LSTM company financial crisis prediction model with feature attention mechanism is shown in Fig. 1. Firstly, the normalized financial data are input to RNN to obtain the prediction data matrix; the output prediction data matrix of RNN is input to the ABC module to perform an optimal global search on initial weights and biases to obtain the new data matrix. The output data matrix of the ABC module is input to the feature attention mechanism layer to get better quality features; the output data of the feature attention mechanism layer is input to the dropout layer to prevent model overfitting; the output data matrix of the dropout layer is passed through Bi-LSTM training sample data and then passed through 1 fully connected layer to get financial crisis prediction results.

### RNN fusion Bi-LSTM network

**Figure 2:**
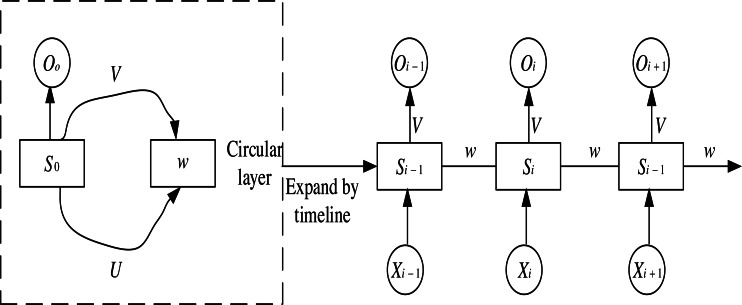
RNN structure diagram.

In RNN networks, the recurrent structure keeps the state value of the hidden neuron at the current moment and inputs it to the neuron as part of the following recurrent input. The input signal of an RNN takes a temporal input. Each layer shares network weights and biases at each input step, significantly reducing the number of parameters to be learned in the network. The structure of an RNN neural network is shown in Fig. 2. The input layer is denoted as $ \left\{ {X}_{0},\ldots ,{X}_{i-1},{X}_{i},{X}_{i+1} \right\} $, the output layer is denoted as $ \left\{ {O}_{0},\ldots ,{O}_{i-1},{O}_{i},{O}_{i+1} \right\} $, and the hidden layer is denoted as $ \left\{ {S}_{0},\ldots ,{S}_{i-1},{S}_{i},{S}_{i+1} \right\} .U,V,W$ are the weight matrixs. At the time *i*, *S*_*i*−1_ the current *X*_*i*_ are used as input, and the result *O*_*i*_ is calculated as output and passed to time *i* + 1 so that each input layer can get the output weight of the previous layer.

**Figure 3:**
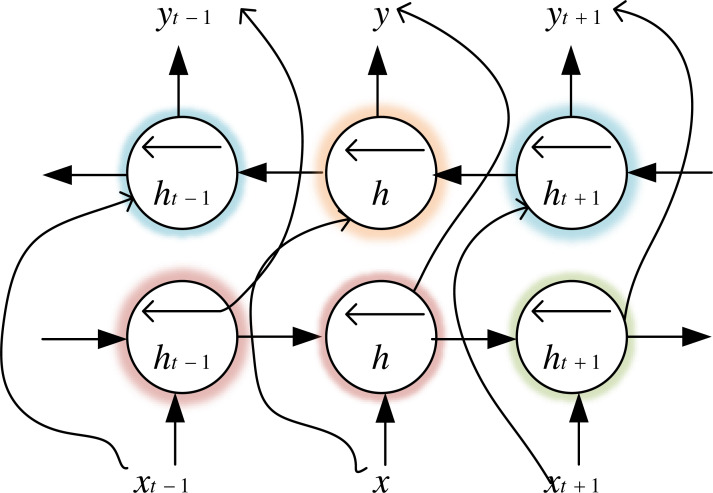
Bi-LSTM structure diagram.

Bi-LSTM is an LSTM variant, which not only has the long-range sequence learning capability of the LSTM model but also further improves LSTM to learn the association relationship between sequence data forward and backwards, which makes the model more advantageous for classification problems. We use financial text data to train the forward LSTM and backward LSTM of BiLSTM, whose structure is shown in Fig. 3. The output vector calculation formula for the time step is shown in (1).

(1) \begin{eqnarray*}{H}_{t}={W}_{hf}^{{^{\prime}}}{h}^{{^{\prime}}}+{W}_{hb}^{{^{\prime\prime}}}{h}^{{^{\prime\prime}}}+{b}_{h}.\end{eqnarray*}

RNN can perform effective feature extraction, but it cannot solve the gradient drop problem, and the prediction accuracy worsens as the series data increases. Bi-LSTM can preserve the long-term dependency between data by merging memory units that can update the previous hidden state. Therefore, combining RNN and Bi-LSTM can solve the long-term dependence problem. Compared with using only the Bi-LSTM network, the RNN and Bi-LSTM network can receive the feature vector matrix of the RNN model to extract the time-series data for financial crisis prediction and capture deeper input data features.

### Feature attention mechanism

**Figure 4:**
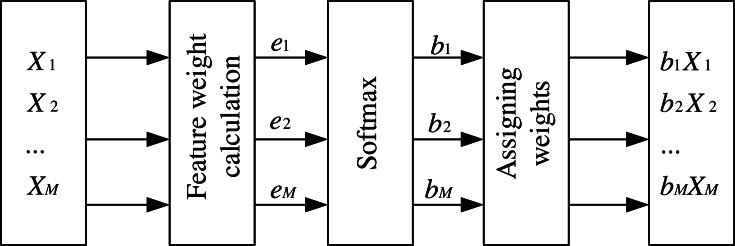
Feature attention mechanism.

The attention mechanism has been applied to various aspects, and its proper use can help the network make better predictions of features. The feature attention mechanism used in this article takes the RNN network output data matrix *X*_1_, *X*_2_, …, *X*_*M*_ as input at the previous moment of the feature time series. It obtains *e*_1_, *e*_2_, …, *e*_*M*_ by calculating the weights of each feature at the current moment, then performs normalization and enhances or weakens the expression of relevant input information according to the attention weights. Then it multiplies the weights obtained at the current moment with the corresponding features and outputs them as *b*_1_*X*_1_, *b*_2_*X*_2_, …, *b*_*M*_*X*_*M*_. The structure is shown in Fig. 4.

### ABC algorithm optimization

ABC algorithm is a search algorithm that simulates group behaviour. It has the characteristics of simple operation, few control parameters, high robustness, and robust searchability for optimal global solutions (Sreenu & Naidu, 2022). It can realize the performance optimization of the network model. In this article, the ABC algorithm is introduced into the company financial crisis prediction model to search the optimal weights and biases of the RNN+Bi-LSTM network. The specific steps of the ABC algorithm include the following parts.

1. Create an RNN network. Denote the weights and biases of the RNN network by the row vector *X*, $X= \left[ w,b \right] $, where *w* is the row vector representation of the network weights with dimension *N* × *M* + *M* × *M* + *M* × *L*, and *b* is the row vector representation of the network biases with dimension *M* + *L*.*N*, *M*, *L*are the number of nerve elements, and the dimension of *X* is denoted as *D*.

2. Initialize the training parameters of the ABC algorithm: the number of food sources *S*_*N*_, the bee colony size *N*_*C*_, the number of employed bees *N*_*e*_, the number of following bees *N*_*o*_, and the maximum number of cycles MCN. Where *S*_*N*_ = *N*_*e*_ = *N*_*o*_, *N*_*c*_ = *N*_*e*_ + *N*_*o*_. Each food source corresponds to an initialized solution *X*.

3. The suitability values for each food source were calculated as follows.

(2) \begin{eqnarray*}fi{t}_{i}= \frac{1}{1+{f}_{i}} .\end{eqnarray*}

4. Enter the loop phase until the MCN is less than the number of iterations.

5. The employed bees perform a domain search for the alternate food source *Vi* from the current food source *Xi* according to ${V}_{i}^{j}={X}_{i}^{j}+rand(-1,1)({X}_{i}^{j}-{X}_{k}^{j})$, where $k\in \left\{ 1,2,\ldots ,{S}_{N} \right\} $ and *k* ≠ *i*, $j\in \left\{ 1,2,\ldots ,D \right\} $. Compare the fitness values of the current food source *X*_*i*_ with the new food source *V*_*i*_ andselect the food source with a higher fitness value according to the greed principle.

6. The odds ratio for food sources is calculated as:

(3) \begin{eqnarray*}{P}_{i}= \frac{fi{t}_{i}}{\sum _{i-1}^{{S}_{N}}fi{t}_{i}} .\end{eqnarray*}

The following bees perform a domain search for new food sources based on the probability formula for the current food source and select the food source with higher fitness based on the greed principle.

7. If the MCN is lower than the number of iterations, perform step 9; otherwise, perform step 5.

8. Initialize the parameters of the RNN+Bi-LSTM network using the solution of the optimal food source, and then start training the RNN+Bi-LSTM network.

9. Evaluate the performance of the improved ABC-RNN+Bi-LSTM network.

## Experiment

The sources of financial data used in this study are the CSMAR database and the Wind database. The specific experimental environment of this article is based on PyTorch deep learning architecture, running on Windows 10 operating system, and the device is NVIDIA Quadro RTX 6000 GPU. The data indicators are shown in Table 1. The sample distribution is shown in Table 2.

**Table 1:** Company operating financial data indicators.

Level 1 indicators	Indicator name
Debt service capacity	Current ratio, quick ratio, cash ratio, equity ratio, cash flow interest coverage multiple, interest earned multiple, long-term gearing ratio, long-term debt-to-equity ratio long-term debt-to-equity ratio
Earnings capacity	Return on net assets, main operating ratio, return on assets, net operating margin, net profit margin on current assets, net profit margin on fixed assets, net profit margin on total assets
Operating capacity	Accounts Receivable Turnover, Current Assets Turnover, Inventory Turnover, Total Assets Turnover, Business Cycle, Accounts Payable Turnover
Development capacity	Basic earnings per share growth rate, net income, total assets growth rate, profit Growth rate of total amount, growth rate of total operating income, growth rate of operating profit
Cash Flow capacity	Net Cash Flow from Operating Activities / Net Income from Operating Activities, Net Cash Flow from Operating Activities Net cash flow from operating activities, cash operating index

**Table 2:** Study sample distribution.

/	ST	Non-ST	Total
Training set	16	48	64
Test set	8	30	38
Total	24	78	102

By calculating the correlation coefficients between various indicators, we can clearly understand which indicators have the most significant impact on the enterprise’s financial risk so that we can take corresponding measures in a targeted manner. After calculating the correlation coefficients among the indicators, we found that the correlation between return on assets, gearing ratio and equity concentration, and financial crisis was the highest, at −0.34, 0.2, and −0.14, respectively. The best prediction result was achieved when the number of RNN nodes was adjusted to 32, the number of Bi-LSTM nodes was 80 and the activation function was a tanh function.

### Analysis of the prediction effect of the ABC-RNN+Bi-LSTM model

The model performs crisis prediction on the training set to obtain the confusion matrix, as shown in Table 3.

**Table 3:** Training set prediction result.

Company category		Predicted result		Number of sample	Accuracy
		ST	Non-ST		
Real situation	ST	29	2	31	96.55%
Real situation	Non-ST	3	29	32	93.6%

**Figure 5:**
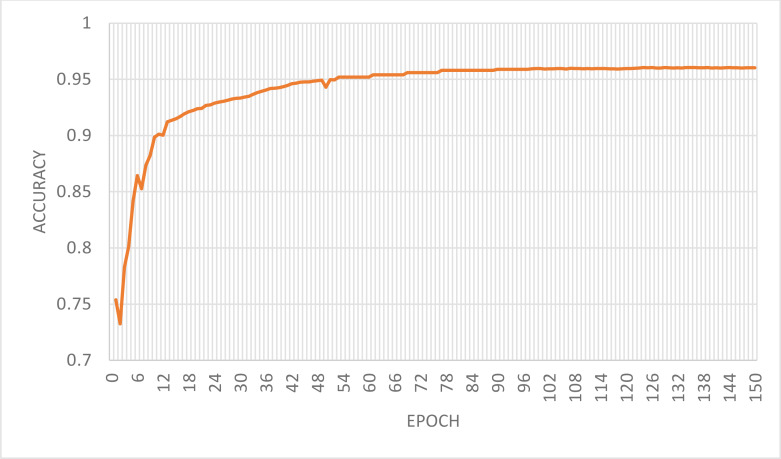
Model classification accuracy graph.

From the prediction results of the training samples, we can see that there are two misclassifications in 31 positive samples and three misclassifications in 32 negative samples, *i.e.,* five misclassifications in 63 samples. It can be deduced that the model’s accuracy is 95.06% and recall is 96.55%, *i.e.,* 29 out of 31 ST companies are detected commonly. The classification accuracy curves of the ABC-RNN+Bi-LSTM+Bi-LSTM model with feature attention mechanism at 150 iterations on the training set are shown in Fig. 5.

It can be seen that the model in this article has been stable after 18 iterations. This is mainly due to the attention mechanism and the ABC algorithm introduced in this article can help the model extract more high-quality features quickly. So the proposed ABC-RNN+Bi-LSTM company financial crisis prediction model with feature attention mechanism has good robustness and generalization ability.

The model performs crisis prediction on the test set to obtain the confusion matrix, as shown in Table 4.

**Figure 6:**
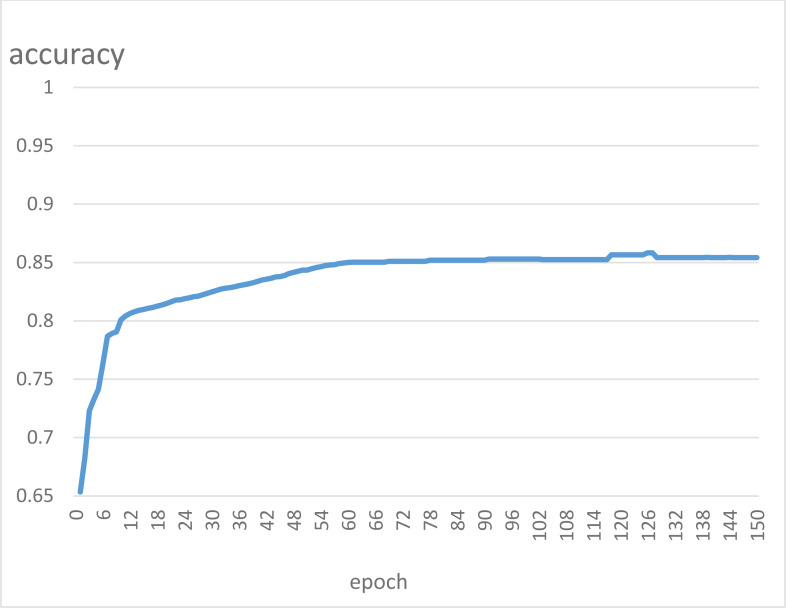
Test set classification accuracy graph.

As can be seen from the prediction results of the test samples, there are seven misclassified companies in 63 samples, and the model’s accuracy is 88.94%. Among them, recall is 88.23%, which means that 30 out of 34 ST companies are detected as normal. Recall measures the model’s ability to identify positive example samples. If a listed company is ST and the model’s judgment is non-ST, it cannot detect that the company is likely to be in crisis. In this sense, a model with a low recall value cannot be called a good model. The classification accuracy curve of the model in this article at 150 iterations on the test set is shown in Fig. 6.

The model in this article still performs well on the test set and compares with its performance on the training set. It can achieve fast convergence and stability overall, although it fluctuates slightly during the iterations.

### Comparison of prediction errors of different models

To verify the improvement of the feature attention mechanism and Bi-LSTM on the prediction ability of RNN, CNN+RNN, GAN+RNN, RNN, and the model in this article are compared with the error experiment. In the error analysis, since the error calculation is trying to know the deviation size of the real point and the predicted point, Mean Square Error(MSE) and Root Mean Square Error (RMSE) can be an excellent measure of the error offset between the real situation and the predicted result. The smaller value indicates the better performance of the model. The error comparison of the four models is shown in Table 5.

**Table 4:** Test set prediction result.

Company category		Predicted result		Number of sample	Accuracy
		ST	Non-ST		
Real situation	ST	30	4	34	88.23%
Real situation	Non-ST	3	26	29	89.65%

**Table 5:** Comparison of errors of different models.

Errors	CNN+RNN	GAN+RNN	RNN	Ours
MSE	2.918 × 10^−5^	3.922 × 10^−5^	2.824 × 10^−5^	2.751 × 10^−5^
RMSE	5.402 × 10^−3^	6.318 × 10^−3^	5.331 × 10^−3^	5.245 × 10^−3^

 It can be seen that the MSE and RMSE of the model in this article are the lowest. This is mainly because the model in this article can deeply capture the input financial data information. In addition, the feature attention mechanism modifies the weight of feature information and further improves the model’s prediction performance. Therefore, compared with other models, the model proposed in this article has apparent advantages in financial crisis prediction.

To verify the enhancement of the feature attention mechanism and RNN on the prediction ability of Bi-LSTM, the prediction accuracy of RNN+Bi-LSTM and Attention+ Bi-LSTM, AR-Bi-LSTM, and the model in this article were compared. The overall MSE and RMSE for the four models are shown in Table 6.

**Table 6:** Prediction error of different models.

Errors	RNN+Bi-LSTM	Attention+ Bi-LSTM	AR-Bi-LSTM	Ours
MSE	6, 048 × 10^−4^	9.579 × 10^−4^	2.961 × 10^−5^	2.751 × 10^−5^
RMSE	7.105 × 10^−2^	3.098 × 10^−2^	5.838 × 10^−3^	5.245 × 10^−3^

It can be seen that: the MSE and RMSE of this article’s model are below the other three models, reflecting the better prediction enhancement effect of RNN and feature attention mechanism for Bi-LSTM, which illustrates the stability and good prediction ability of this article’s model. In summary, the feature attention mechanism and RNN better the LSTM’s prediction enhancement for the financial crisis than using it alone.

## Conclusion

Major businesses are quickly emerging due to the ongoing promotion of sustainable development techniques, reviving the market economy to its full potential. However, as each business grows, the high demand for technological iteration, poor ability to service debt, and erratic cash flow increase the risk of a future financial collapse. This study suggests a financial crisis prediction model based on the ABC-RNN+Bi-LSTM algorithm in sustainable development that assists businesses in anticipating financial risks. To extract more time-series feature vectors from financial data using the RNN, Bi-LSTM, and feature attention mechanism. Additionally, the feature attention mechanism extracts better depth features from economic data. Finally, the ABC algorithm is introduced to optimize the weights and biases of the RNN. The experiment shows that this model’s prediction accuracy and recall rate in the test set are 88.94% and 88.23%, respectively, which can achieve accurate financial crisis prediction. Next, we will consider reducing the model parameters to achieve more efficient financial crisis prediction. The limitation of the proposed strategy was the difficulty in training the model. We intend to improve this limitation, ultimately increasing the model’s accuracy.

##  Supplemental Information

10.7717/peerj-cs.1287/supp-1 Supplemental Information 1 Code used for the implementation

10.7717/peerj-cs.1287/supp-2 Supplemental Information 2 Data set used for the project (downloaded from Kaggle)

## Additional Information and Declarations

### Data Availability

The following information was supplied regarding data availability: The project code and data sets used to implement the project are available in the Supplemental Files. The data sets are available at the UCI Machine Learning Repository: https://archive.ics.uci.edu/ml/datasets/Dow+Jones+Index.
