# Supplementary figures and images for: Design of a corporate financial crisis prediction model based on improved ABC-RNN+Bi-LSTM algorithm in the context of sustainable development
Source: PeerJ Comput Sci. 2023 Apr 26;9:e1287. doi: 10.7717/peerj-cs.1287 (PMC10280568; doi:10.7717/peerj-cs.1287)

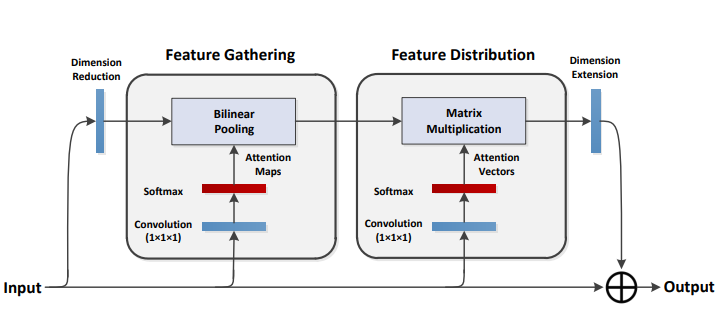

Supplement: Supplemental Information 1 [file peerj-cs-09-1287-s001.zip › code/attention/img/A2.png]

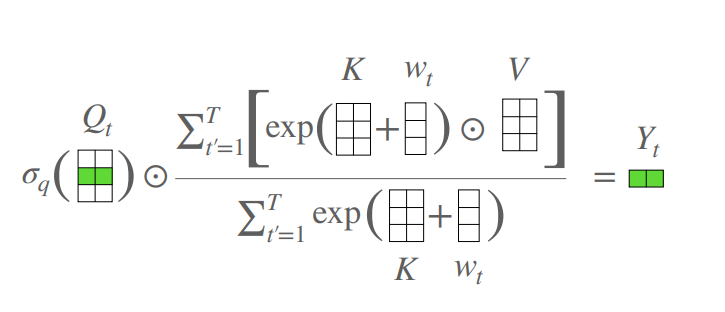

Supplement: Supplemental Information 1 [file peerj-cs-09-1287-s001.zip › code/attention/img/AFT.jpg]

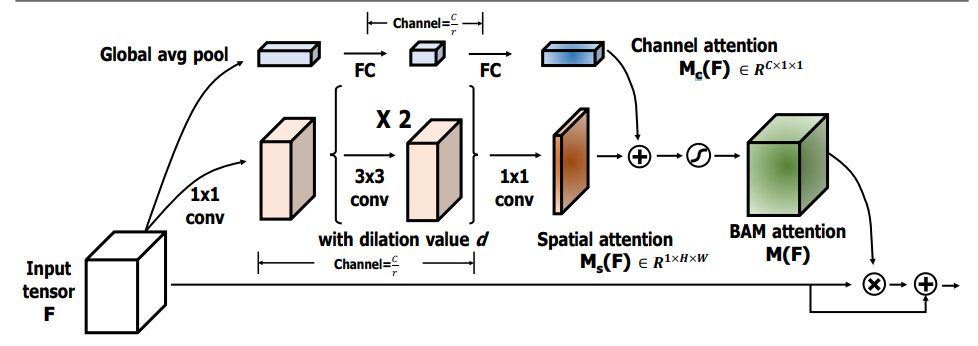

Supplement: Supplemental Information 1 [file peerj-cs-09-1287-s001.zip › code/attention/img/BAM.png]

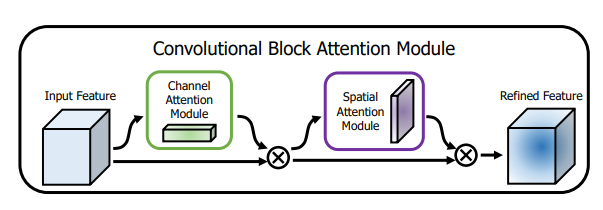

Supplement: Supplemental Information 1 [file peerj-cs-09-1287-s001.zip › code/attention/img/CBAM1.png]

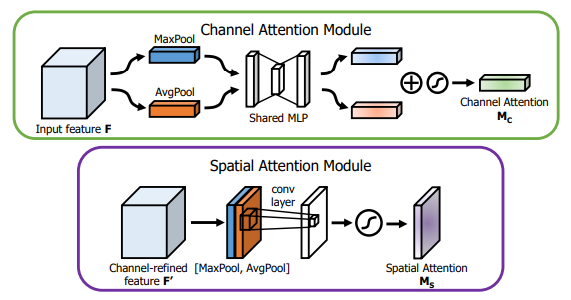

Supplement: Supplemental Information 1 [file peerj-cs-09-1287-s001.zip › code/attention/img/CBAM2.png]

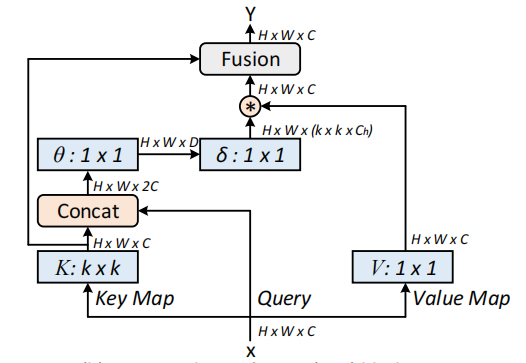

Supplement: Supplemental Information 1 [file peerj-cs-09-1287-s001.zip › code/attention/img/CoT.png]

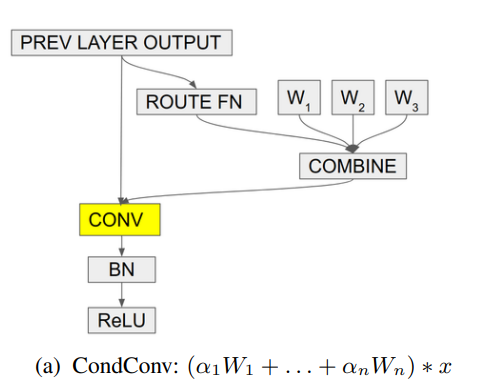

Supplement: Supplemental Information 1 [file peerj-cs-09-1287-s001.zip › code/attention/img/CondConv.png]

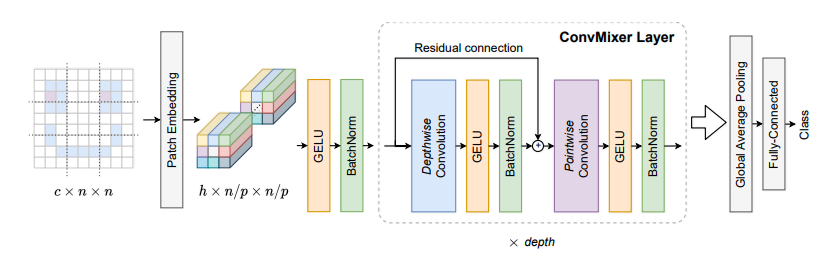

Supplement: Supplemental Information 1 [file peerj-cs-09-1287-s001.zip › code/attention/img/ConvMixer.png]

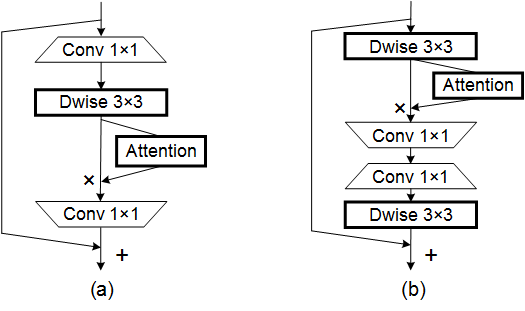

Supplement: Supplemental Information 1 [file peerj-cs-09-1287-s001.zip › code/attention/img/CoordAttention.png]

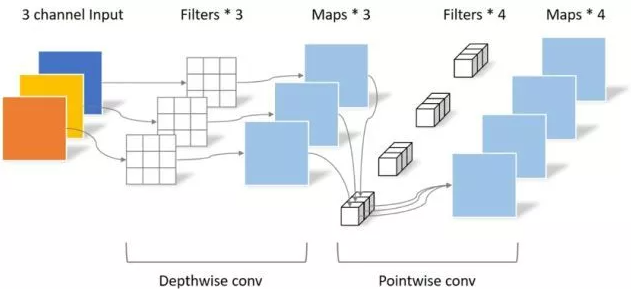

Supplement: Supplemental Information 1 [file peerj-cs-09-1287-s001.zip › code/attention/img/DepthwiseSeparableConv.png]

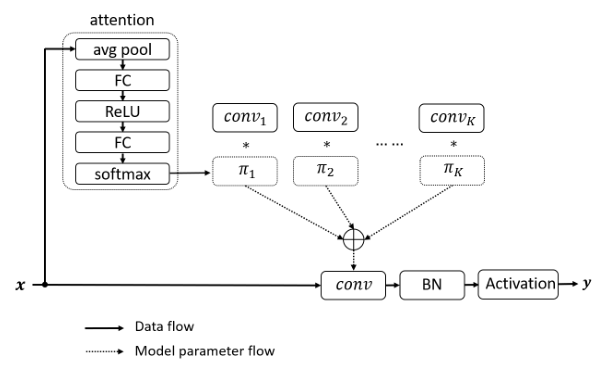

Supplement: Supplemental Information 1 [file peerj-cs-09-1287-s001.zip › code/attention/img/DynamicConv.png]

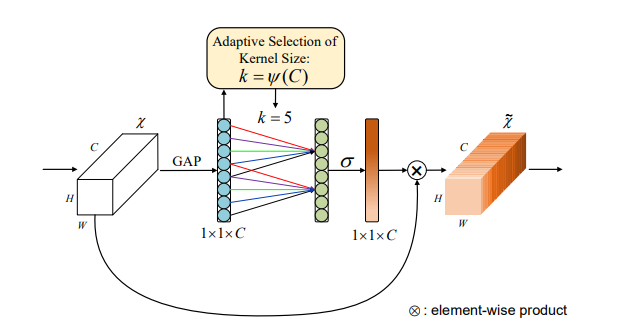

Supplement: Supplemental Information 1 [file peerj-cs-09-1287-s001.zip › code/attention/img/ECA.png]

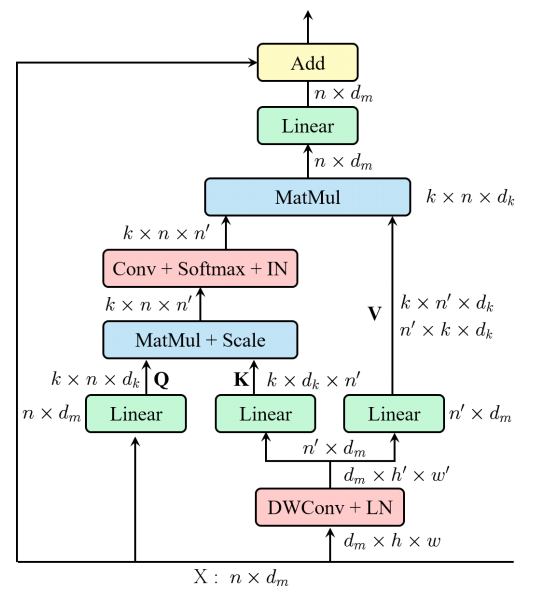

Supplement: Supplemental Information 1 [file peerj-cs-09-1287-s001.zip › code/attention/img/EMSA.png]

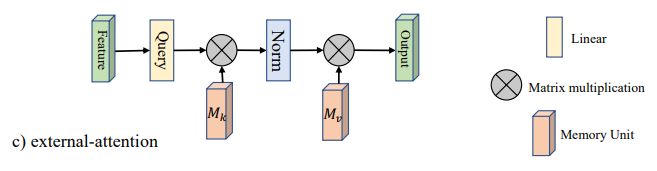

Supplement: Supplemental Information 1 [file peerj-cs-09-1287-s001.zip › code/attention/img/External_Attention.png]

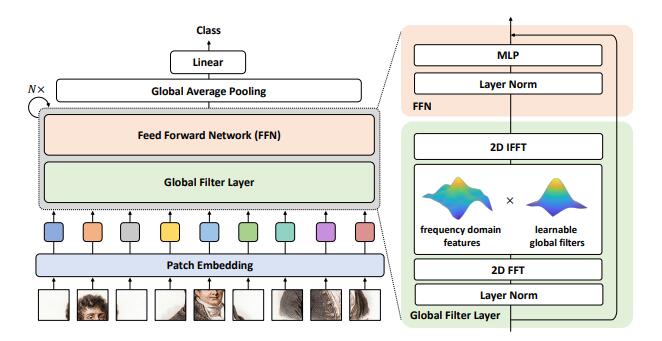

Supplement: Supplemental Information 1 [file peerj-cs-09-1287-s001.zip › code/attention/img/GFNet.jpg]

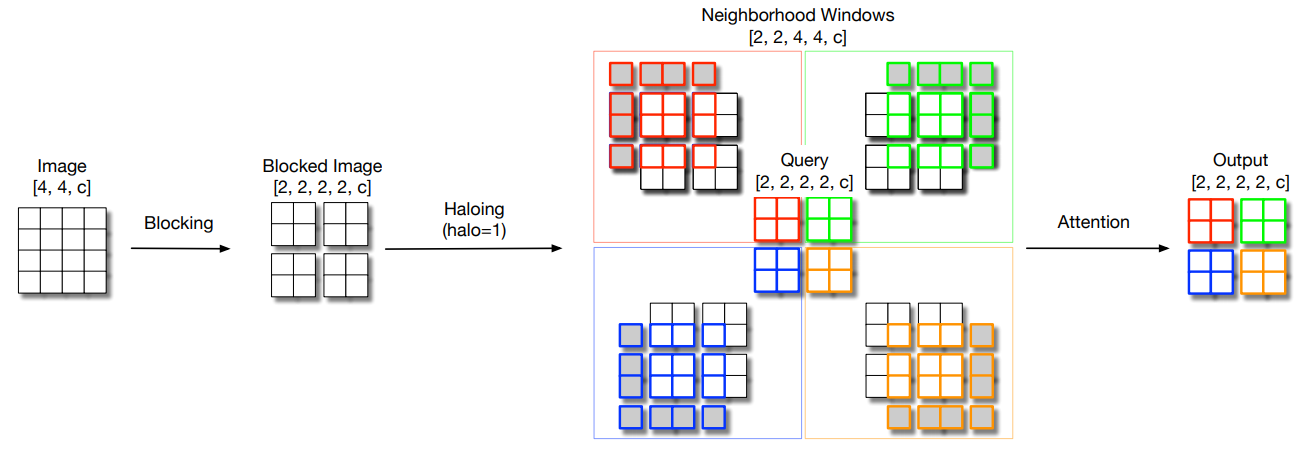

Supplement: Supplemental Information 1 [file peerj-cs-09-1287-s001.zip › code/attention/img/HaloNet.png]

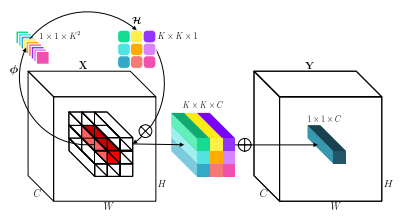

Supplement: Supplemental Information 1 [file peerj-cs-09-1287-s001.zip › code/attention/img/Involution.png]

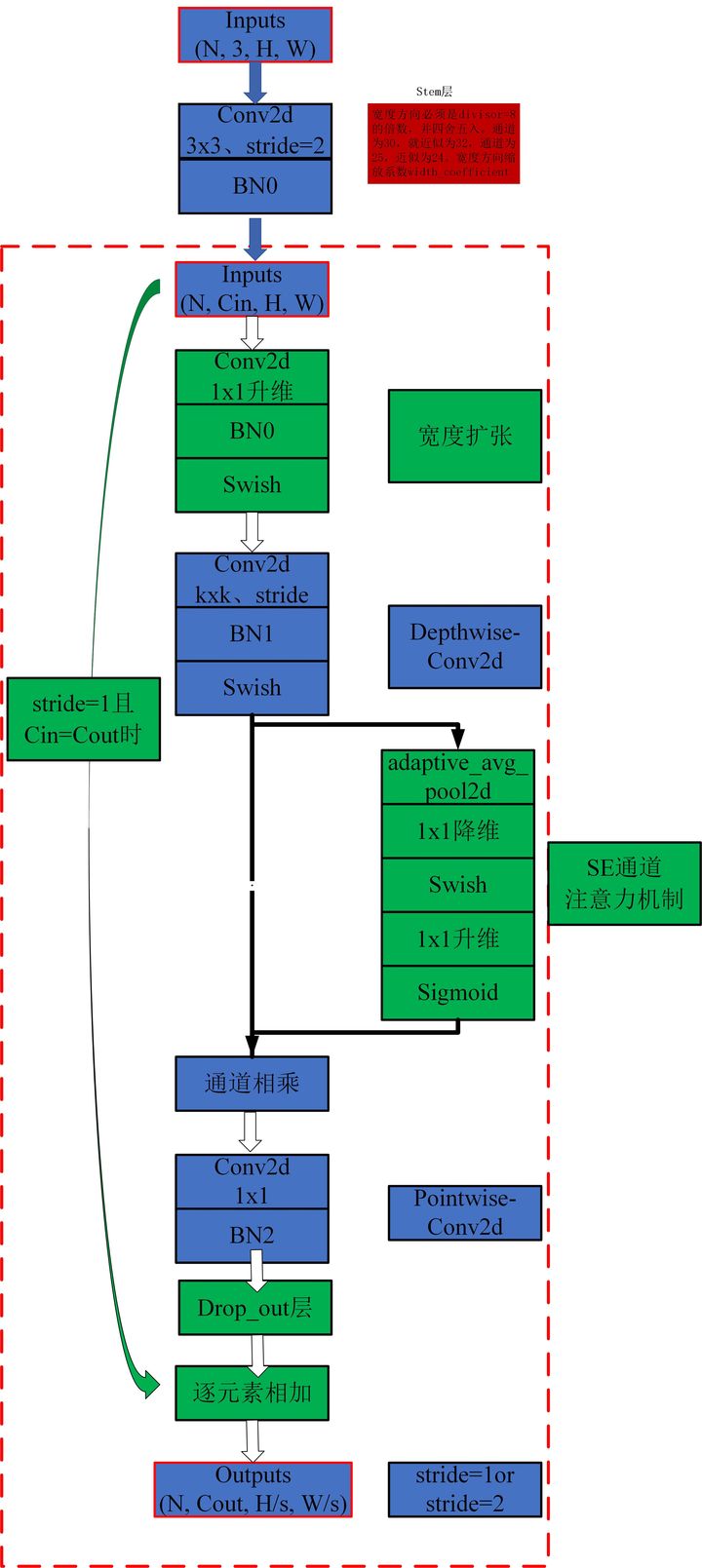

Supplement: Supplemental Information 1 [file peerj-cs-09-1287-s001.zip › code/attention/img/MBConv.jpg]

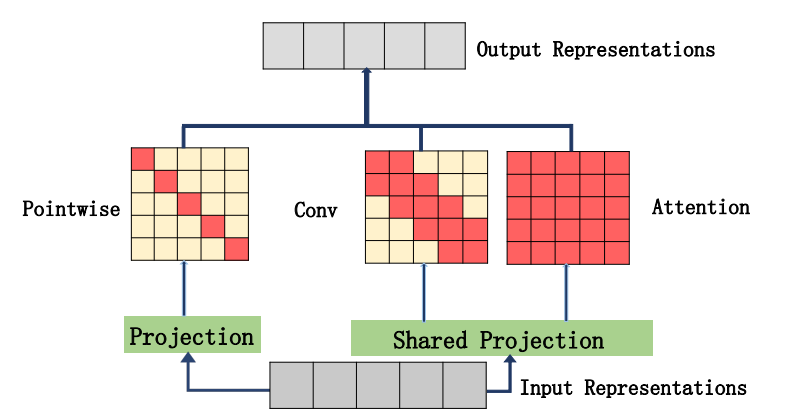

Supplement: Supplemental Information 1 [file peerj-cs-09-1287-s001.zip › code/attention/img/MUSE.png]

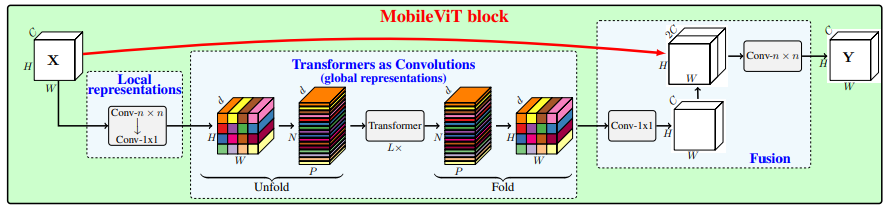

Supplement: Supplemental Information 1 [file peerj-cs-09-1287-s001.zip › code/attention/img/MobileViTAttention.png]

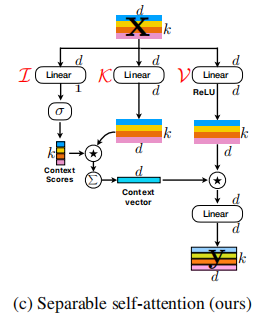

Supplement: Supplemental Information 1 [file peerj-cs-09-1287-s001.zip › code/attention/img/MobileViTv2.png]

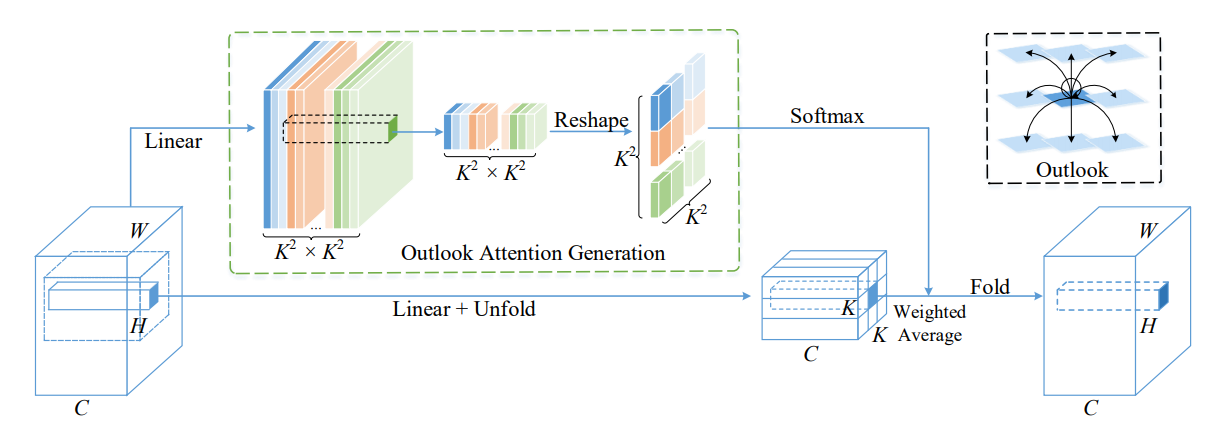

Supplement: Supplemental Information 1 [file peerj-cs-09-1287-s001.zip › code/attention/img/OutlookAttention.png]

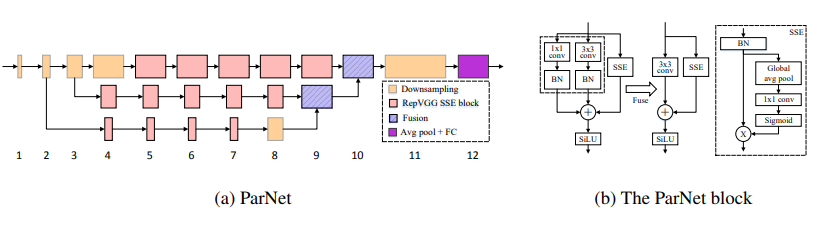

Supplement: Supplemental Information 1 [file peerj-cs-09-1287-s001.zip › code/attention/img/ParNet.png]

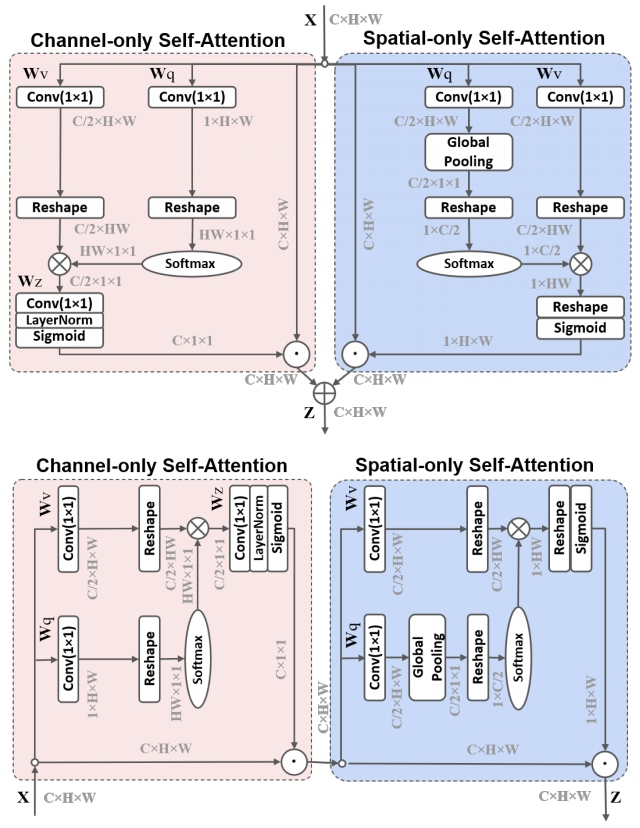

Supplement: Supplemental Information 1 [file peerj-cs-09-1287-s001.zip › code/attention/img/PoSA.png]

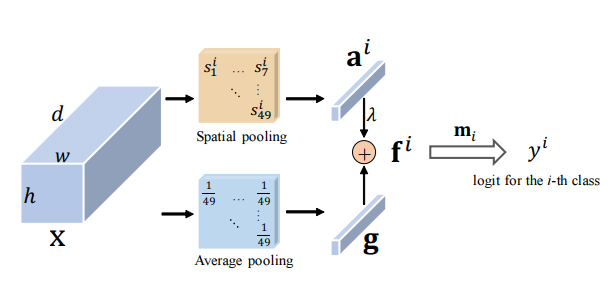

Supplement: Supplemental Information 1 [file peerj-cs-09-1287-s001.zip › code/attention/img/ResAtt.png]

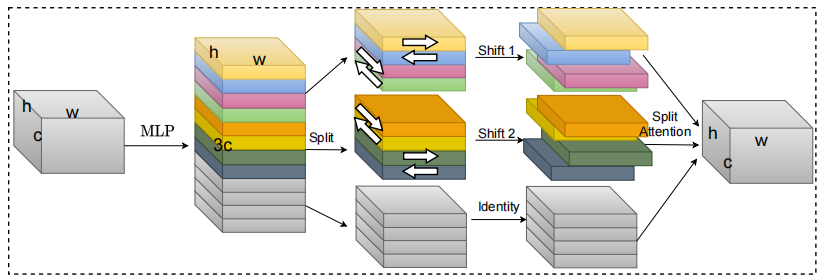

Supplement: Supplemental Information 1 [file peerj-cs-09-1287-s001.zip › code/attention/img/S2Attention.png]

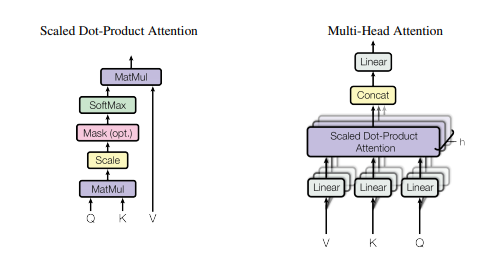

Supplement: Supplemental Information 1 [file peerj-cs-09-1287-s001.zip › code/attention/img/SA.png]

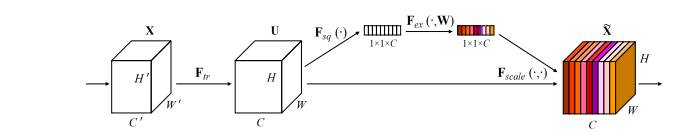

Supplement: Supplemental Information 1 [file peerj-cs-09-1287-s001.zip › code/attention/img/SE.png]

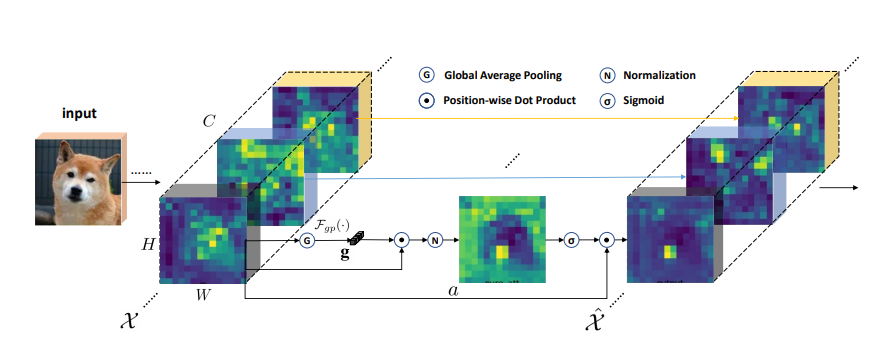

Supplement: Supplemental Information 1 [file peerj-cs-09-1287-s001.zip › code/attention/img/SGE.png]

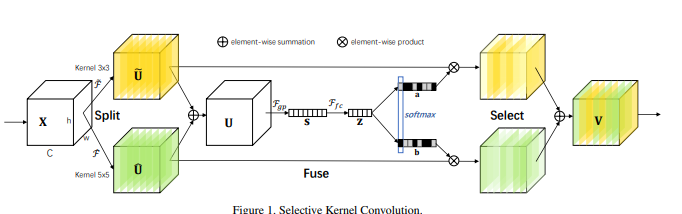

Supplement: Supplemental Information 1 [file peerj-cs-09-1287-s001.zip › code/attention/img/SK.png]

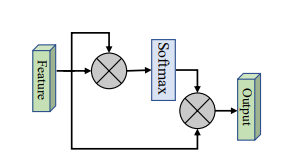

Supplement: Supplemental Information 1 [file peerj-cs-09-1287-s001.zip › code/attention/img/SSA.png]

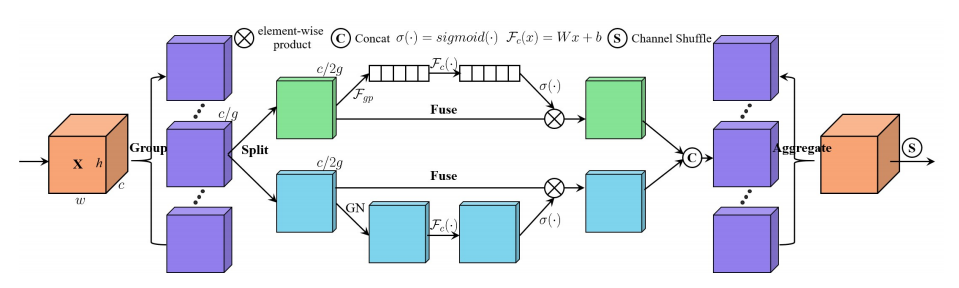

Supplement: Supplemental Information 1 [file peerj-cs-09-1287-s001.zip › code/attention/img/ShuffleAttention.jpg]

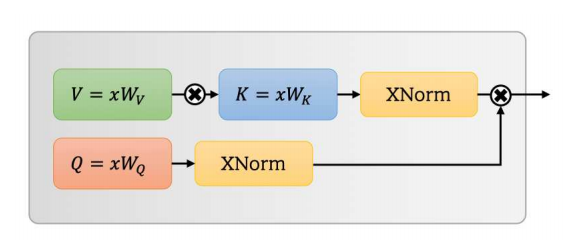

Supplement: Supplemental Information 1 [file peerj-cs-09-1287-s001.zip › code/attention/img/UFO.png]

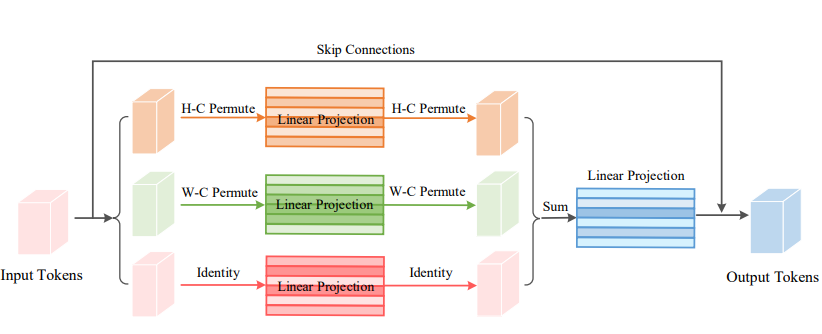

Supplement: Supplemental Information 1 [file peerj-cs-09-1287-s001.zip › code/attention/img/ViP.png]

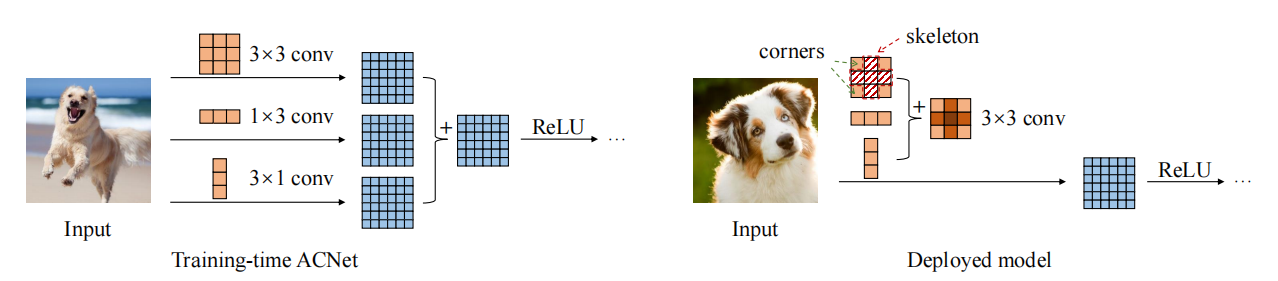

Supplement: Supplemental Information 1 [file peerj-cs-09-1287-s001.zip › code/attention/img/acnet.png]

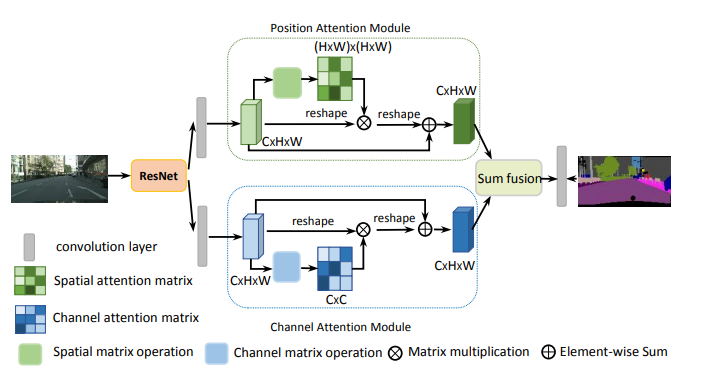

Supplement: Supplemental Information 1 [file peerj-cs-09-1287-s001.zip › code/attention/img/danet.png]

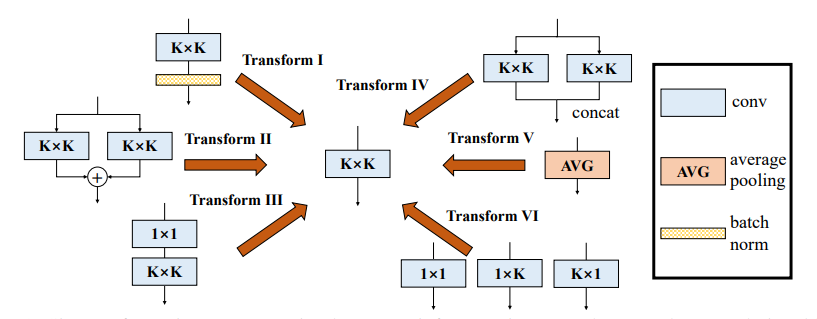

Supplement: Supplemental Information 1 [file peerj-cs-09-1287-s001.zip › code/attention/img/ddb.png]

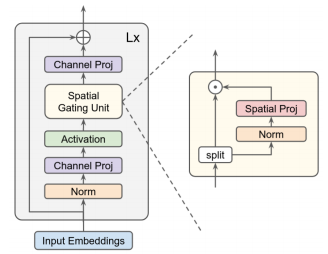

Supplement: Supplemental Information 1 [file peerj-cs-09-1287-s001.zip › code/attention/img/gMLP.jpg]

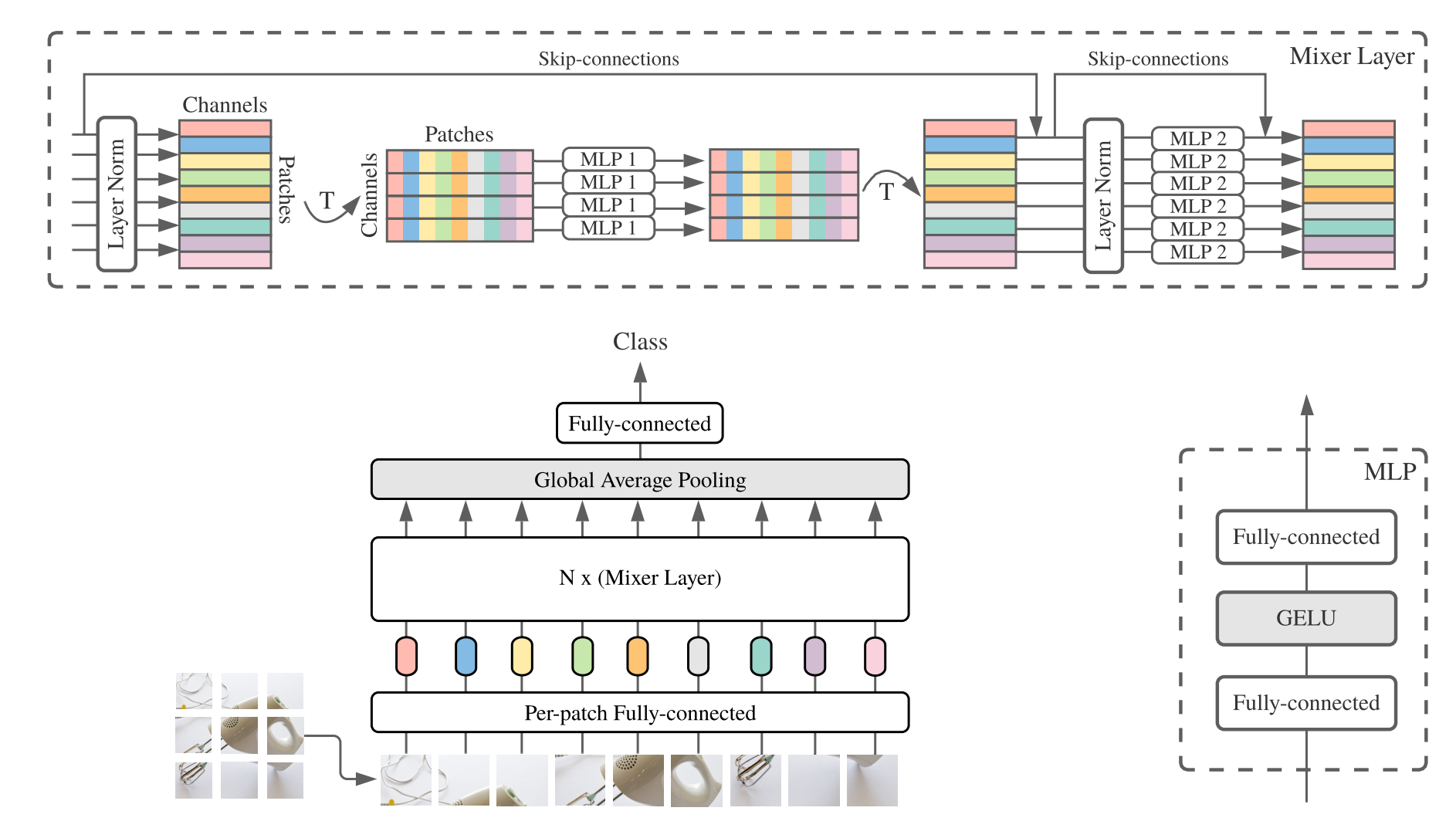

Supplement: Supplemental Information 1 [file peerj-cs-09-1287-s001.zip › code/attention/img/mlpmixer.png]

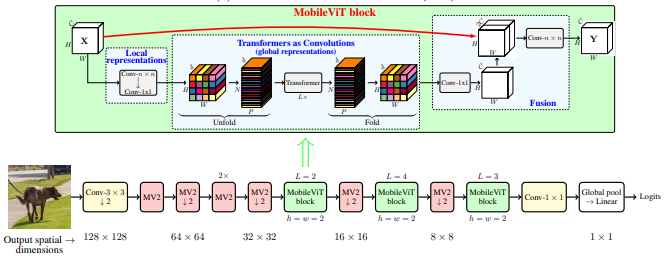

Supplement: Supplemental Information 1 [file peerj-cs-09-1287-s001.zip › code/attention/img/mobileViT.jpg]

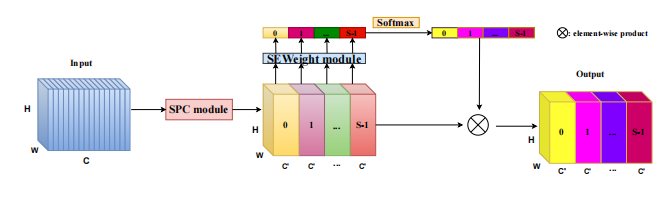

Supplement: Supplemental Information 1 [file peerj-cs-09-1287-s001.zip › code/attention/img/psa.png]

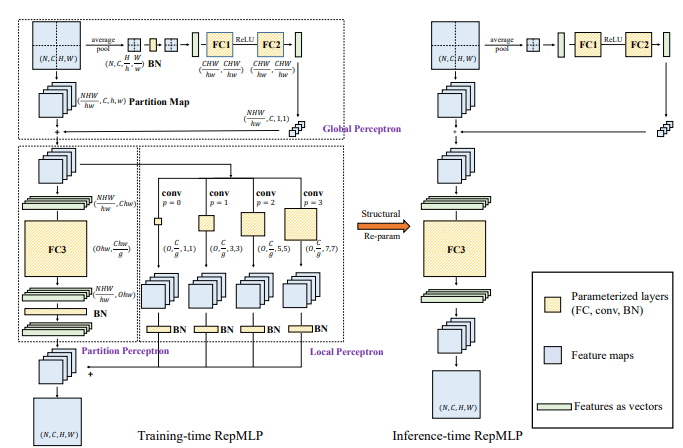

Supplement: Supplemental Information 1 [file peerj-cs-09-1287-s001.zip › code/attention/img/repmlp.png]

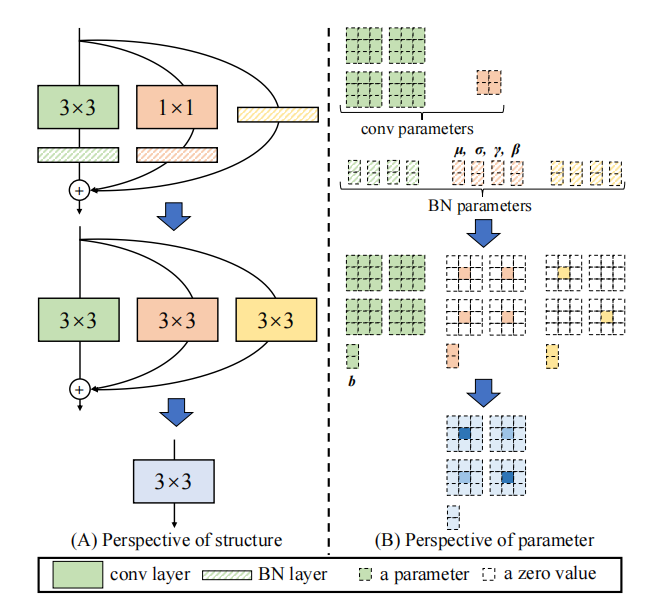

Supplement: Supplemental Information 1 [file peerj-cs-09-1287-s001.zip › code/attention/img/repvgg.png]

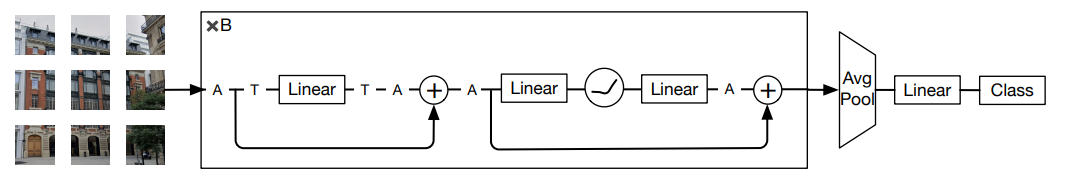

Supplement: Supplemental Information 1 [file peerj-cs-09-1287-s001.zip › code/attention/img/resmlp.png]

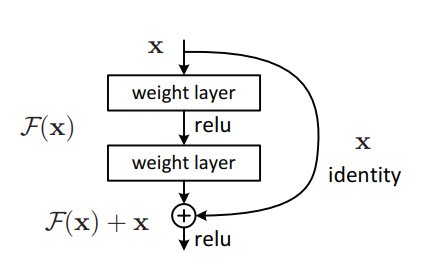

Supplement: Supplemental Information 1 [file peerj-cs-09-1287-s001.zip › code/attention/img/resnet.png]

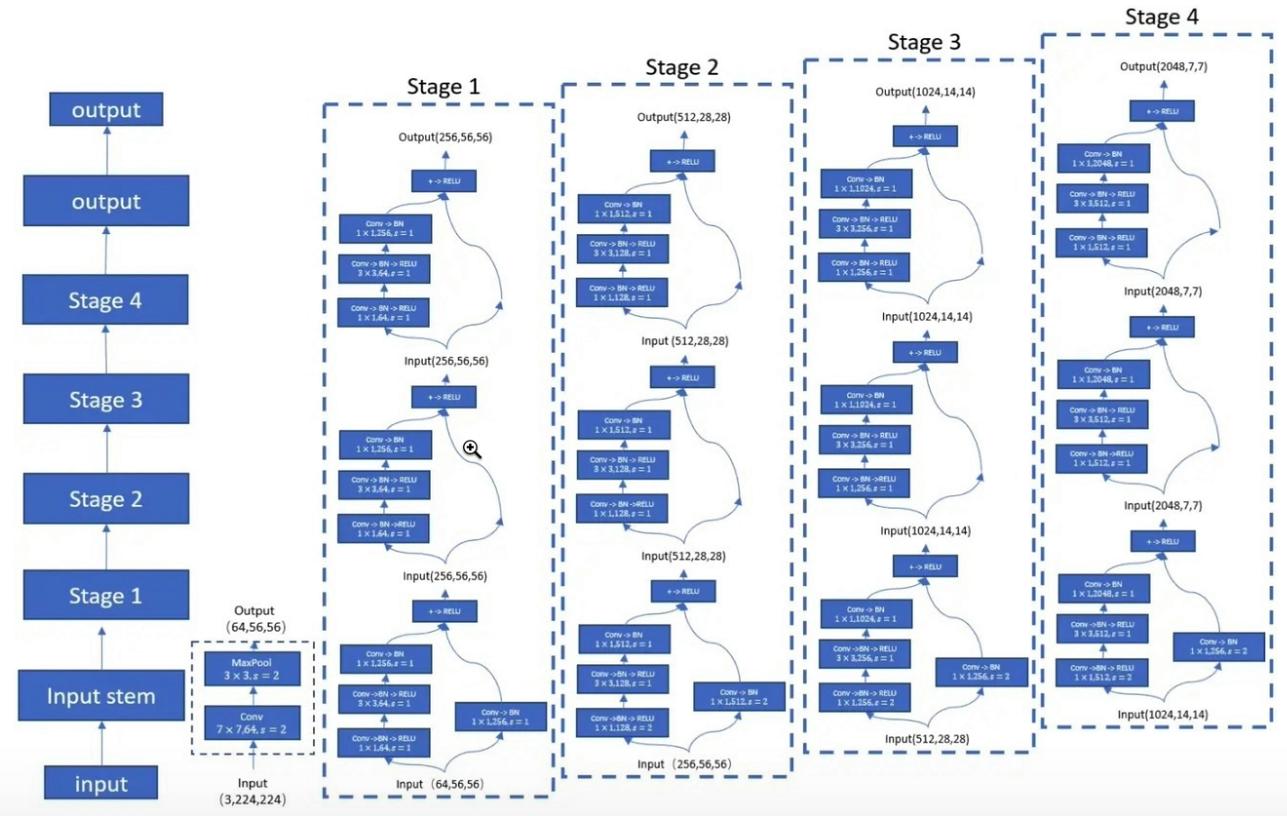

Supplement: Supplemental Information 1 [file peerj-cs-09-1287-s001.zip › code/attention/img/resnet2.jpg]

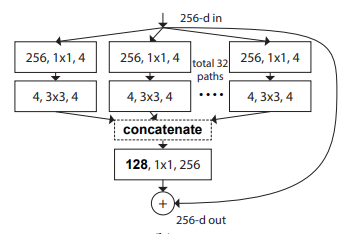

Supplement: Supplemental Information 1 [file peerj-cs-09-1287-s001.zip › code/attention/img/resnext.png]

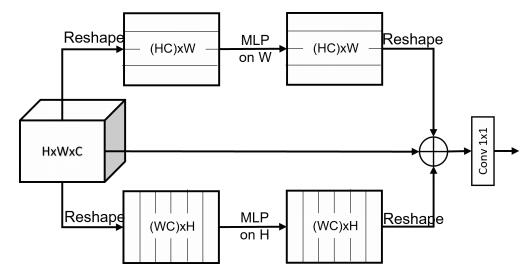

Supplement: Supplemental Information 1 [file peerj-cs-09-1287-s001.zip › code/attention/img/sMLP.jpg]

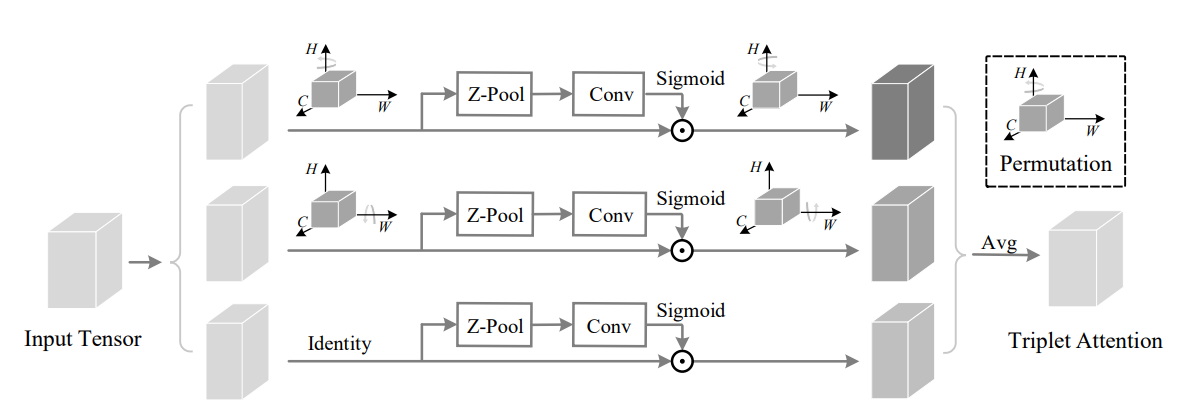

Supplement: Supplemental Information 1 [file peerj-cs-09-1287-s001.zip › code/attention/img/triplet.png]
